# Effectiveness of psychological techniques in dental management for children with autism spectrum disorder: a systematic literature review

**DOI:** 10.1186/s12903-022-02200-7

**Published:** 2022-05-06

**Authors:** Ismail Nabil AlBhaisi, Marisa Shanthini Thomas Santha Kumar, Anissha Engapuram, Zaleha Shafiei, Ahmad Shuhud Irfani Zakaria, Shahida Mohd-Said, Colman McGrath

**Affiliations:** 1 Department of Restorative Dentistry, Faculty of Dentistry, Universiti Kebangsaan Malaysia (The National University of Malaysia), Jalan Raja Muda Abdul Aziz, 50300 Kuala Lumpur, Malaysia; 2 Department of Craniofacial Diagnostics and Biosciences, Faculty of Dentistry, Universiti Kebangsaan Malaysia (The National University of Malaysia), Jalan Raja Muda Abdul Aziz, 50300 Kuala Lumpur, Malaysia; 3 Department of Family Oral Health, Faculty of Dentistry, Universiti Kebangsaan Malaysia (The National University of Malaysia), Jalan Raja Muda Abdul Aziz, 50300 Kuala Lumpur, Malaysia; 4 Applied Oral Sciences and Community Dental Care, Faculty of Dentistry, The University of Hong Kong, Prince Philip Dental Hospital, 34 Hospital Road, Sai Ying Pun, Hong Kong

**Keywords:** Dental management, Autism spectrum disorders, Dental care, Dental setting, Behaviour modification, Thinking differences, Learning differences

## Abstract

**Background:**

A rise in the reported numbers of children with Autism Spectrum Disorder (ASD) highlights the need for dental practitioners to be more familiar with the treatment approaches for these special needs children to ensure comfortable, well-accepted and efficient management while in dental office.

**Aim:**

This paper aimed to acquire a deeper understanding of some of the innovative and best approaches to managing children with ASD in dental settings.

**Design:**

A systematic literature search was performed in PubMed, Scopus, Web of Science, Cochrane databases, and grey literature based on the PRISMA 2020 statement, using main keywords such as: ‘management’, ‘dental’, ‘children’, and ‘Autism Spectrum Disorder’. Original full-text papers including randomised controlled trials (RCT) and all other designs of non-randomised controlled studies (NRS) reporting relevant intervention studies in English were included without any publication time limit. The quality of the evidence found eligible for the review were then assessed using the ROB-2 and ROBINS-I tools. Subsequently, the details of management interventions and impact of treatment approaches were compared and discussed.

**Results:**

Out of the 204 articles found, 109 unrelated articles were excluded during the initial screening. The full papers of remaining 28 were retrieved and only 15 (7%) articles were eligible to be reviewed; eight RCTs with ‘some concerns’ and ‘high risk’ categories particularly concerning their randomisation design, and seven NSRs with ‘serious’ to ‘critical’ bias largely due to confounding factors.

**Conclusion:**

Our review found inconclusive evidence on the strength of recent psychological and non-pharmacological approaches used to manage children with ASD in dental settings. Small sample size and lack of a control group in certain studies affected the strength of evidence and credibility of the findings. Nevertheless, this review shared informative details on some innovative approaches for better understanding of the management of children with ASD for dental professionals.

## Highlights

- Explores deeper knowledge and understanding of psychological approach for managing children with ASD in a clinical dental setting.
- Highlight the impact of such intervention on dental anxiety, the level of children’s cooperation, and the success of the implementation of dental procedures, which will help the dentists to meet and treat children with ASD according to their individual needs.
- Discuss the evidence in favour of the use of behaviour management in reducing anxiety and enhancement of cooperation in children with ASD at the dental setting.

## Introduction

Children with autism spectrum disorders (ASD) commonly face anxiety and fear when undergoing dental treatment, as manifested via difficult behaviours and uncooperative reactions [1, 2]. The special congestive profile of autistic children and the specific process related to the response and adaptability to the surrounding environment exhibit a wide spectrum of behaviour alterations [3, 4]. Children with ASD often show prominent characteristics of aggressiveness, unresponsiveness, lack of attention, and the presence of other medical signs that may compromise the dental treatment plan [1]. In addition to ASD, the term autism spectrum condition (ASC) has also been used to emphasise on the biomedical diagnosis of the learning and thinking differences in affected individuals [5]. This issue further complicates the fact that several studies have found that the oral health of children with ASD is worse than that of typical children due to lack of awareness among the dental community in how to increase a caregivers’ oral hygiene practices for their children, difficulty in accessing dental care facilities, and the knowledge and attitude of dental professionals towards the children [6, 7].

Communication between the child and dental team in clinic can be very difficult or restricted [8] if there is no standard protocol to manage these children especially while being treated. Thus, the dental team must attempt different ways of communications, behavioural management, and pharmacological management to control the child [9, 10]. Altered behaviours among autistic children and their tendencies of self-injury further increase the risk of unresponsiveness or even trauma during dental treatment and prevent the clinicians from performing comprehensive dental treatment. In such scenarios, more aggressive techniques such as Protective Stabilization Board (papoose) or general anaesthesia may be required [6], and these may not be well-received by patients and caregivers. Alternatively, some studies have focused on the effectiveness of specific behavioural or psychological approaches either on oral care or as a communication-aided intervention [11, 12], general strategies of ASD management in a dental office [13] and visual aid approaches (visual pedagogy) using either printed or electronic materials [14, 15].

So far, the effectiveness of more recent pharmacological and psychological (non-pharmacological) strategies to improve the dental management of children with ASD has not been reported systematically and are not well known to most dental professionals. Therefore, this systematic literature review aimed to evaluate the effectiveness of available reported behaviour management and modification strategies for children with ASD to overcome the anxiety and discomfort associated with the treatment in dental clinics. This review may provide the necessary evidence for clinical guidelines on the management of dental anxiety, the acceptance, success rates, and impact of each approach with the aim of improving the oral health status and wellness of the children.

## Materials and methods

This systematic literature review was conducted in compliance with the “Preferred Reporting Items for Systematic Reviews and Meta-Analysis” (PRISMA 2020 statement). It is registered under the “International Prospective Register of Systematic Reviews” (CRD42021273415), and received approval for conduct by the research ethics committee (UKM PPI/111/8/JEP-2020-757).

### Search strategy and definitions

The PICO strategy was utilised in answering the research questions: What is the impact of special techniques in dental management for children with autism spectrum disorder on their cooperation while undergoing treatment in dental clinic? The study population (P) of interest was children with ASD within the range of 2–18 years old who were receiving interventions (I) including special dental management techniques in the dental setting as well as other intervention aimed at improving the success and cooperation of children while receiving dental treatment. The results from this survey were compared (C) with healthy children, children with any other disabilities, or another ASD group receiving other intervention(s). The expected outcome (O) from the intervention was the improvement in cooperation during dental procedures as rated by dental professionals or caregivers, improvement in the behaviour scale, and a decreased level of anxiety.

### Selection criteria

The search strategy was carried out in the following database: Scopus, Web of Science, PubMed, and Cochrane, as well as grey literature searches included Google Scholar and hand-search the reference lists of all included articles and relevant literature reviews. The core keywords included (management) AND (child*) AND ("Autism Spectrum Disorder" OR ASD OR autism OR "Asperger syndrome") AND (dental). The Medical Subject Headings, MeSH (https://meshb.nlm.nih.gov/search) was also used to identify words and phrases from articles of interest (Table 1). No time limit was set in this search.

**Table 1:** Search strategy for literature

Database	Search string	Limits/Inclusion
SCOPUS	(TITLE-ABS-KEY (“Autism Spectrum Disorder") OR TITLE-ABS-KEY (ASD) OR TITLE-ABS-KEY (autism) OR TITLE-ABS-KEY ("Autistic Disorder")) AND TITLE-ABS-KEY (child*) AND TITLE-ABS-KEY (dental) AND TITLE-ABS-KEY (management) AND ( LIMIT-TO (PUBSTAGE, "final")) AND (LIMIT-TO (DOCTYPE, "ar")) AND (LIMIT-TO ( LANGUAGE, "English"))	Language: English Document: Articles Stage: Final
Web of Science	[TS = (child*) AND TS = ("Autism Spectrum Disorder" OR ASD OR autism OR "Asperger syndrome") AND TS = (management) AND TS = (dental)]	Language: English Timespan: All years Indexes: SCI-EXPANDED, SSCI, A&HCI, CPCI-S, CPCI-SSH, BKCI-S, BKCISSH, ESCI
PubMed	(management) AND (child*) AND ("Autism Spectrum Disorder" OR ASD OR autism OR "Asperger syndrome") AND (dental)	Language: English Full text
Cochrane	(management) AND (child*) AND ("Autism Spectrum Disorder" OR ASD OR autism OR "Asperger syndrome") AND (dental)	Language: English

The inclusion criteria were: original full-text papers for studies involving children of 2–18 years old, randomised controlled trials (RCT) or all designs of non-randomised controlled study (NRS), i.e. non-RCT, interventional study, studies with comparative groups, interrupted time series study, cohort study, controlled before-and-after study, and case series (uncontrolled longitudinal study). Furthermore, the full-text article must be written in the English language and report the impact of the intervention in the form of behaviour scales or cooperation rate. Studies that focused only on the perceptions and concerns of the caregivers or those with insufficient information on the outcome were excluded from the review.

### Study selection

The articles obtained from the search were exported into Microsoft Excel. The list of articles was screened for replicates and their relevance to the study title. Any duplicates or non-ASD-related articles were rejected. Two researchers (MS and SE) screened the titles and abstracts of all the retrieved full-text articles to filter out those that were not relevant to the research question. If there was some disagreement on the relevance of the articles between the two researchers, it would be resolved through discussion with the other three reviewers (S.M-S., Z.S, and I.N.B.).

### Data extraction

For each of the included articles, the following information was obtained: general characteristics (authors, year of publication, title, and study design), the sample size of subjects, comparative groups, assessment tools used in the study, dental procedures done in each study, type of management or techniques as intervention, outcome measures (e.g. improvement in the anxiety and behaviour scores, changes before and after intervention related to improvement in achievement in planned dental procedure to be implemented), and lastly key findings.

### Risk of bias assessment

The reviewers assessed the risk of bias of the included studies independently. Studies with NRS designs were evaluated using the ROBINS-I “Risk Of Bias In Non-randomised Studies-of Interventions” and the studies were rated with the same coding of the data extraction process. The seven domains of ROBINS-I assessed are risk of bias arising from (confounding, selection of participants, classification of interventions, deviations from intended interventions, missing data, measurement of outcomes, selection of the reported result) respectively. In addition, the bias of the RCT studies was evaluated using version 2 of the Cochrane Risk-of-bias tool for randomised trials (ROB-2) and the data in the table were generated using the Excel tool provided by the same team. The five domains of ROB-2 assessed are risk of bias arising from (randomization process, deviation from the intended interventions, missing outcome data, measurement of outcomes, and selection of the reposted results) respectively. Criteria for reaching the overall judgements for studies included in both (ROB-2 or ROBINS-I) tools were performed in compliance with the guidelines for each tool [16, 17]. Meanwhile, the inter-evaluator reliability was calculated using Kappa statistics.

## Results

### Study selection

**Fig. 1:**
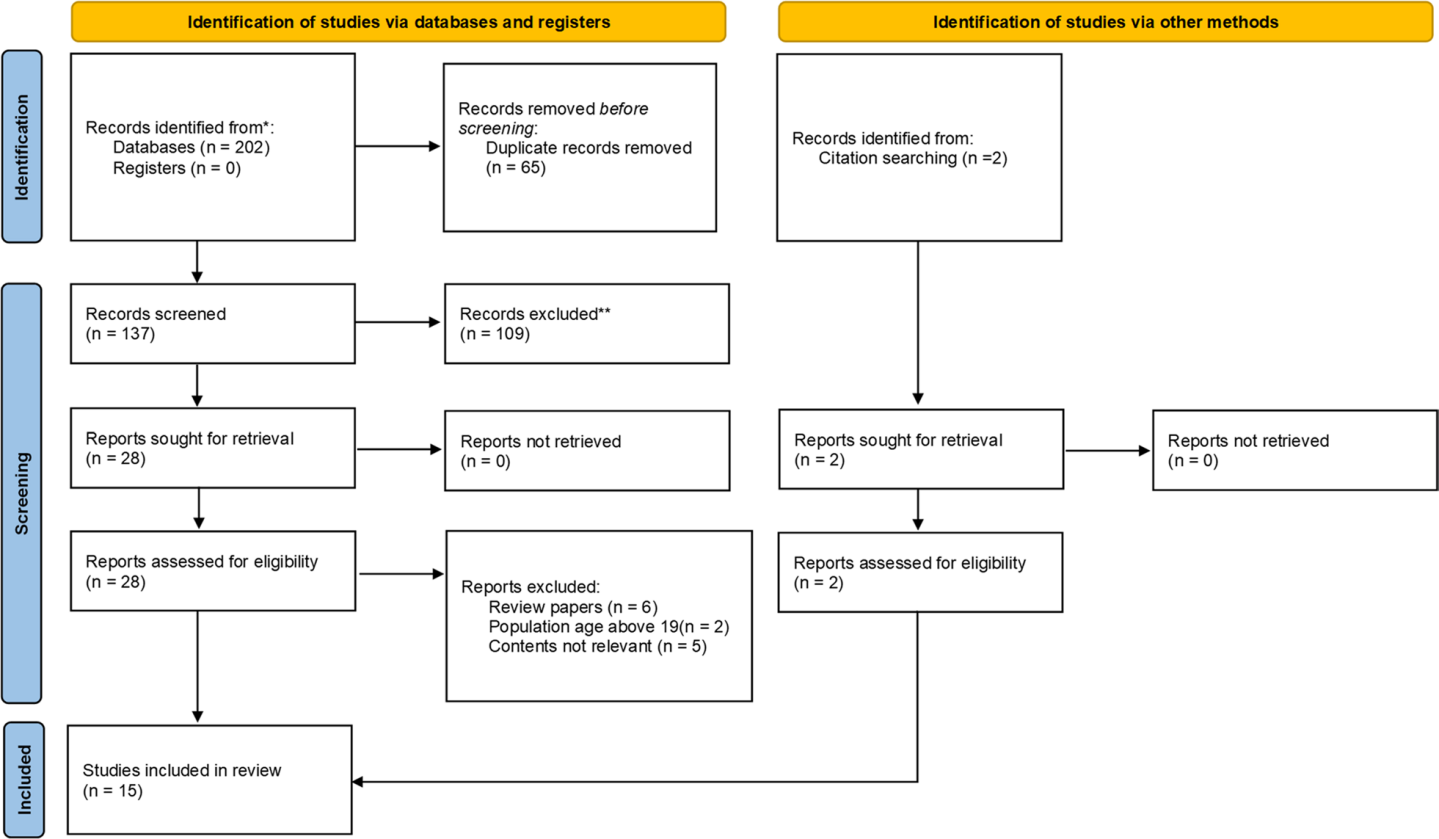
Summary of literature selection process for systematic review

Final search date was 1st January 2022. The initial search retrieved 202 papers from four databases; 65 were found to be duplicates. One hundred and nine papers were excluded due to the irrelevance of titles and/ or abstracts (Agreement between reviewers was high, K = 0.92). Fifteen were excluded based on full-text ratings (Agreement between reviewers was high, K = 0.86). Additionally, two papers were added scanning the references lists of eligible papers. The step-by-step search and selection strategy is shown in Fig. 1 using the PRISMA template for systematic literature review [18].

### Characteristics of the studies

Of the 15 articles selected, 8 were RCT [19–26] and 7 were NRS; of which three were interrupted time series study (ITSSs) [27–29]. All the included studies were organised according to the year of publication and intervention approach. The total number of children involved were 904, of which 862 were children with ASD. The age of the children ranged from 2–18 years with a predominance of male children across the studies. The range of the time interval was two months in between of analysis (Table 2).

**Table 2:** Description of reviewed studies

Studies	Design and assessment tool	Children involved	Comparative groups	Dental procedures received
Lefer et al. 2019 [27]	Interrupted time-series study Cooperation of children in dental assessment	52 ASD children and adolescents: 3–19 years old 7 females, 45 males	No control group	Clinical oral assessment
Zink et al. 2018 [19]	Randomised clinical trial Number of dental appointments needed to perform the procedure	40 children with ASD: 9–15 years old 2 females, and 38 males	Two groups: Application group: (2 females, 18 males) PECS: (20 males)	Dental prophylaxis using low-speed handpiece Topical fluoride application
Hidayatullah et al. 2018 [28]	Interrupted time-series study Customised engagement checklist on 10 stages of the procedure	13 children with ASD: 5–18 years old 2 females, 11 males	One ASD group	Dental examination
Nilchian et al. 2017 [20]	Randomised clinical trial Cooperation of children in clinical examinations	40 children with ASD: 6–12 years old 3 females, 37 males	20 children in each group	Fluoride therapy
Tounsi et al. 2017 [31]	Retrospective cohort study The success of dental examination	168 children with ASD: 4–18 years old 28 females, 140 males	No control group	Dental examination only
Murshid et al. 2017 [33]	Cross-sectional non-randomised controlled trial study Parents’ evaluation and procedures performed	40 children with ASD: 5–9 years old 10 females, 30 males	No control group	Oral examinations Prophylaxis, and topical fluoride applications
Nelson et al. 2017 [30]	Retrospective cohort study Successful dental examination	168 children with ASD: 4–18 years old 29 females, 139 males	No control group	Dental examination
AlHumaid et al. 2016 [32]	Retrospective cohort study Frankl behaviour rating scale and dental procedures completed	44 children with ASD: 5–18 years old 14 females, 30 males	22 in each group	70% received dental treatment: Cleanings (50%) Restorative treatment (18%) Extractions (2%)
Marion et al. 2016 [21]	Randomised controlled trial study Caregivers’ preference via questionnaire	40 children with ASD and their caregivers: 18 years old 6 females, 34 males	No control group	No treatment given
Mah & Tsang 2016 [22]	Randomised control trial Cooperation of children in dental assessment	14 children with ASD: 3–8 years old 14 males	Two ASD group Tell-show-do with visual pedagogy = 7 Tell-show-do only, N = 7	Dental examination
Cagetti et al. 2015 [29]	Interrupted time-series study Acceptance rate of the treatment	83 children with ASD: 6–12 years old 18 females, 65 males	Three groups undergoing same intervention: 6–7 years 8–9 years 10–12 years	Children underwent four stages: An oral examination (stage 1) A professional oral hygiene session (stage 2) Sealants (stage 3) If necessary, a restorative treatment (stage 4)
Cermak et al. 2015 [23]	Crossover randomised trial Physiological stress and anxiety, measured by electrodermal activity (EDA)	44 children: 6–12 years old 16 females, 28 males	22 ASD children 22 non-ASD children	Oral examination Prophylaxis (dental cleanings) Fluoride application
Isong et al. 2014 [24]	Randomised controlled trial study Venham Anxiety and Behaviour Scales	80 children with ASD: 7–17 years old 15 females, 65 males	Each group had 20 children Four groups: Group A: Usual care Group B: A DVD video of a typically developed child having a dental appointment was used for video peer modelling Group C: Sunglass-style video eyewear was used to view a favourite movie during a dentist visit Group D: Video of peer modelling plus video goggles	Extra-oral and intra-oral examinations with radiographs Scaling (if needed) Prophylaxis Application of fluoride varnish
Orellana et al. 2014 [25]	Non-randomised control trial Cooperation of children in dental assessment	72 persons with ASD: 4–41 years old 24 females, 38 males	38 children and 34 adults	Clinical oral assessment
Lowe & Lindemann 1985 [26]	Randomised controlled trial study Successful oral examination	40 children: Mean age 12.5 years old 12 females, 28 males	20 ASD children 20 non-ASD children	Extra-oral and intra-oral examination with radiographs

In most studies, the cooperation of children during dental assessment was the most frequent tool used to assess the impact of the approach used [20, 22, 25, 27, 29, 30], followed by the success of oral examination [26, 31, 32], caregivers’ preference [21, 33], number of dental appointments to perform the planned procedure [19], customised engagement checklist [28], and lastly, behaviour rating scales such as Frankl [32], electrodermal activity (EDA) [23], and Venham [24].

### Outcomes of the intervention approach

In this systematic review, the main outcome was determined by the improvement in the child’s cooperation during dental procedures as rated by dental professionals or caregivers. Another main outcome was the improvement in the behaviour and decrease in the anxiety level of the children in the dental setting. Accordingly, the measures of effect for the outcomes reported in the studies were the increase in the success rate or completion of dental procedure, i.e., the increase in the number of components achieved in a dental visit, and/ or improvement on the behaviour rating scales.

All the approaches were evaluated according to the planned procedure. Most of the studies focused on the clinical oral assessment and examination as main dental procedures to be assessed [22–31, 33]. Some other studies focused on more advanced procedures such as dental prophylaxis and topical fluoride application [19, 20, 23, 24, 29, 33]. Only two studies focused on dental treatment such as restorative treatment and extractions [29, 32] (Table 3).

**Table 3:** Intervention techniques for managing children with ASD

Studies	Description of method of intervention		Outcomes of intervention
	Control	Test	
Lefer et al. 2019 [27]	No control groups	çATED app showing pictures of dental examination using iPad	65.4% percentage individuals showed improved compliance during oral assessment Time interval: Eight months (evaluation at two-, four-, six-, and eight-month)
Zink et al. 2018 [19]	Picture exchange communication system by flashcards with pictures of routine at dental office	A communication app consists of representative images accompanied by written and corresponding audio comments describing the phases of the dental treatment	Decrease in number of dental visits and attempts to acquire each skill between two groups (3/5) respectively Time interval: Not applicable
Hidayatullah et al. 2018 [28]	No control group	(Applied Behaviour Analysis) ABA based management methods using image cards	Improvement in behavioural stages for 11 children One child was able to complete all stages Time interval: Treatment was conducted four times at one-week intervals for a month
Nilchian et al. 2017 [20]	Standard examination without any intervention	Visual pedagogy (set of colouring pictures illustrated dental examination steps)	Cooperation during fluoride therapy increased in the case group (6/1) respectively Cooperation in the control group did not increase in most stages Both groups presented the same findings in opening of mouth and showing the teeth, or entering the office, and sitting in the chair or examination with mirror Time interval: Practices for 8 weeks
Tounsi et al. 2017 [31]	No control group	Dental desensitisation	77% of ASD children were successfully examined within 1 to 2 visits in compared to 88% by the fifth visit 12.5% could not receive dental examination Time interval: Two visits only
Murshid et al. 2017 [33]	No control group	A children’s book preparing children and their parents for the first dental visit	47.5% of ASD children acted positively during the dental procedure 37.5% showed positive effect on the behaviour of children according to their parents’ evaluation Time interval: 6 months (evaluation at week-1 and 4 months)
Nelson et al. 2017 [30]	No control group	Progressive desensitisation with individualised reinforcements. (The child is gradually exposed to glimpses from the dental setting that cause anxiety, and rewards as positive reinforcement.)	Minimal threshold examination (MTE) was achieved for 77.4% of all children within 1 to 2 visits and 87.5% in 5 visits or less Desensitisation was effective in achieving an MTE for most children Time interval: 5 dental visits
AlHumaid et al. 2016 [32]	Standard Behavioural Guidance Techniques (SBGTs) including tell-show-do (TSD), voice control (VC), nitrous oxide (NO), passive restraint, and active restraint (AR)	D-TERMINED Programme used the familiarisation process through the philosophy of repetitive tasking	D-TERMINED programme group had significantly lower referral rate compared to the SBGTs group Frankl scale showed significant improvement in the behaviour of test group in compared to SBGTs group 52% of participants showed improvement in behaviour Time interval: Mean number of dental visits: 2–6
Marion et al. 2016 [21]	No control group	Dental stories available via different media (paper, tablet computer, and computer)	Nine (64%) caregivers found the dental story useful Two (14%) caregivers found the aid was only helpful for themselves Time interval: 6-month until follow-up survey was completed
Mah & Tsang, 2016 [22]	TSD (tell-show-do) only	Visual pedagogy with TSD method	Cooperation level during dental treatment increased Completed more steps in final appointment Decreased time required to achieve child cooperation Lower level of behavioural distress Time interval: 3 weeks
Cagetti et al.2015 [29]	No control group	Visual aid: Sketch of the steps of the four planned dental procedures: (Oral examination, dental hygiene appointment, fissure sealants, and restorative procedure)	77 subjects (92.8%) overcame both stage 1 and 2 6 subjects (7.2%) refused stage 3 3 subjects (7.2%) refused stage 4 Time interval: 1.5 months
Cermak et al. 2015 [23]	Regular dental environment (RDE) – existing practise and setting	Sensory adapted environment (SADE) applied in the dental environment in three aspects, i.e. visual, auditory, and tactile: Visual: Shading the windows with curtains and turning off the dental chair Auditory: playing rhythmic music lamp Tactile (deep pressure): papoose board looks like a butterfly with its wings	Significant decrease in electrodermal activity (EDA) in SADE compared to RDE Effect size of the SADE vs RDE (0.23ASD/0.29 non-ASD) Time interval: 3–4 months
Isong et al. 2014 [24]	Usual care (Group A)	Group B: A DVD video of a typically developed child having a dental appointment was used for video peer modelling Group C: Sunglass-style eyewear was used for children to view a favourite movie during a dentist visit Group D: Video of peer modelling plus video goggles	Between visits 1 and 2, the mean anxiety and behaviour scores decreased significantly among subjects within groups C and D compared to others Time interval: 6 months (evaluated baseline and at the end of the study)
Orellana et al. 2014 [25]	No control group	TEACCH-Based Approach (Treatment and Education of Autistic and related Communication-handicapped Children)	The mean number of steps achieved significantly increased in children between pre- and post-intervention Time interval: 4 weeks (evaluated baseline and at the end of the study)
Lowe & Lindemann, 1985 [26]	Negative reinforcements (e.g. “you won’t get lunch”), if positive reinforcements (e.g. rewards) failed	Positive reinforcements, with tell-show-do (TSD)	Using Positive reinforcements (85% ASD/ 90% Non-ASD) was successfully examined on first visit Negative reinforcement was used among 8 ASD and 2 Non-ASD children ASD/Non-ASD (10/18) patients underwent bitewing radiographs Time interval: NA

A variety of approaches have been proposed to improve the management of children with ASD. So far, visual pedagogy appeared as the most common approach [28]. It can be in the form of printed materials that demonstrate the dental settings and procedures in a colourful way to the parents and/ or children [28, 33]. Digital-based visual pedagogy on mobile devices or iPad applications was found to confer a more superior impact on the outcome compared to the printed materials [19, 21, 29]. One study in this review focused on the use of digital visual pedagogy as the main approach [27]. Also, the standard clinical dental examinations without any visual pedagogy approach were compared with examinations with use of printed materials [20], and use of video materials (DVD, video goggles, and video modelling) [24]. Meanwhile, the desensitisation programme led to an improvement of the children as seen on the Frankl behaviour scale [30, 31], especially when compared to the standard behaviour guidance approaches that included tell-show-do (TSD), voice control (VC), passive restraint, active restraint (AR), and pharmacological options such as nitrous oxide (NO) [32]. The positive reinforcements supported with TSD showed superiority when compared with negative reinforcements [26]. Finally, another impressive approach was the “Treatment and Education of Autistic and related Communications Handicapped Children” (TEACCH) that included all the communication strategies such as TSD and visual pedagogy to educate and manage the children with ASD [25] (Table 3).

### Risk of bias assessment

The characteristics of the studies were assessed individually to evaluate the outcomes and effects of the interventions using the specific tools based on the study design (Table 2).

**Fig. 2:**
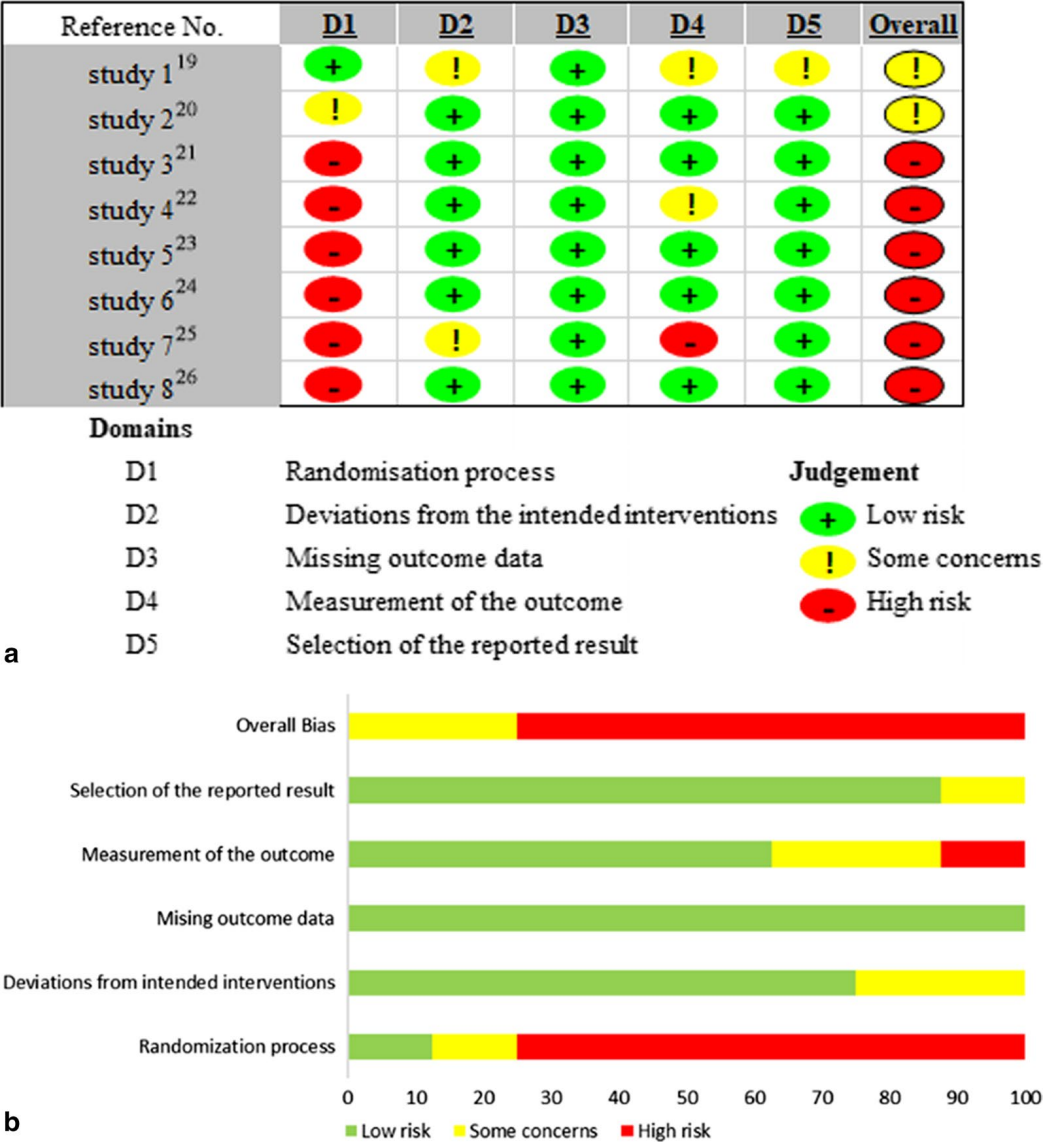
Risk of bias assessment **a** Traffic light plot of RCTs using the ROB-2 tool. **b** Summary plot of RCTs using the ROB-2 tool

The reviewers assessed the quality of the eight RCTs using Version 2 of ROB-2 [19–26] (Fig. 2). Six studies were judged as having a high risk of bias [21–26] and two with a moderate risk of bias [19, 20].

**Fig. 3:**
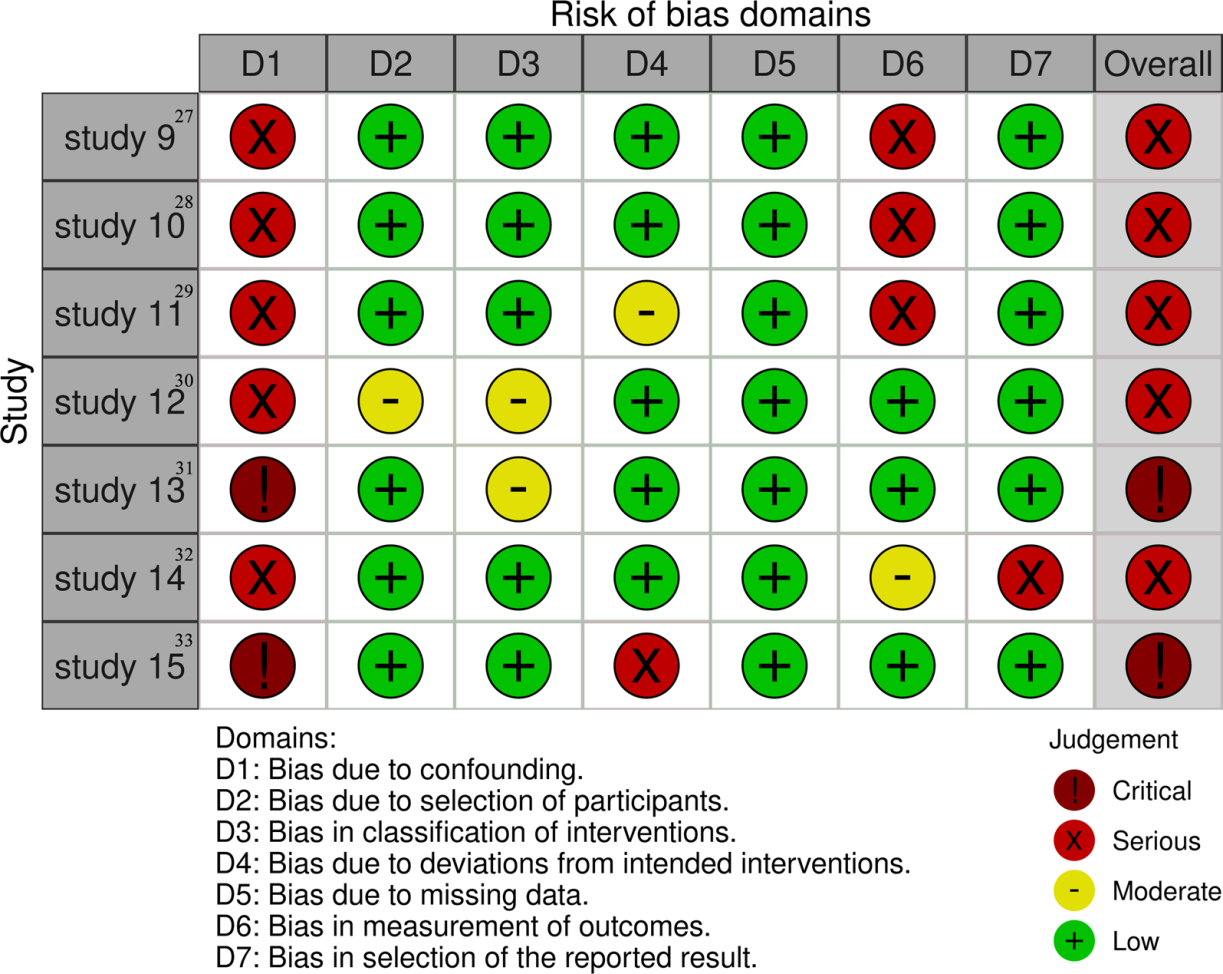
Risk of bias assessment of non-randomised studies of intervention (NRSI) using the ROBINS-I tool

The seven NRS studies were assessed using the ROBINS-I tool. Five studies were judged as having a serious risk of bias [27–30, 32] and two with critical risk of bias [31, 33] (Fig. 3).

## Discussion

In this review, we took into consideration the substantial difference between behavioural management and behavioural modification in line with the proper definition of dental management for children with ASD. Behavioural management is a central component of paediatric dentistry while behavioural modification focused on dealing with the problem, challenges, or avoidance behaviours to ease dental treatment and perform the planned procedures [34].

In the included studies, various approaches were used to improve the management of children with ASD. The significance of behavioural modification in the dental setting was also highlighted. Many behavioural scales have been developed and validated to measure the level of behaviour and its association to anxiety and fear among children. Frankl behavioural rating scale is one of the most widely used. It categorises the children's behaviour into four groups based on their attitude and cooperation during dental treatment [35]. Additionally, the Venham scale was developed to rate the level of anxiety and uncooperativeness of the child towards dental stress [36].

In this review, most of the studies focused on visual pedagogy since it was one of the conventional approaches to manage children in the dental setting. Visual pedagogy in the form of printed material such as dental stories or coloured books about dental treatment can help the parents and/ or children to adapt faster to the dental environment [28, 33]. Additionally, digital visual pedagogy materials including mobile devices/ iPad applications such as çATED app and Picture Exchange communication system (PECS) were more impactful than the printed materials [19, 21, 27, 29]. The standard examination showed a clear improvement with the introduction of printed materials, especially during fluoride therapy [20]. Meanwhile, video materials such as DVDs, video goggles, and video modelling also improved the mean anxiety and behavioural scores [24].

Furthermore, the desensitisation programme was associated with an improvement in the Minimal Threshold Examination (MTE) and behavioural level of the children, as manifested by an improvement in children’s cooperation during the dental examination [30, 31], especially among children with moderate ASD. Desensitisation programmes, such as D-TERMINED are built on familiarisation and repetitive tasking of specific procedures, also known as the Sensory Adapted Environment (SAE) that was developed from the Applied Behaviour Analysis theory (ABA). The desensitisation programme was found to be superior to the standard behavioural guidance approach that included communication strategies, restraint, and even the pharmacological options as nitrous oxide (NO) [32].

Next, the positive reinforcements supported by TSD also showed an improvement in cooperation during dental examination compared to negative reinforcements [26]. Finally, one of the most impressive approaches, “TEACCH” that incorporated all the communication strategies such as TSD, visual pedagogy approaches was beneficial in the management of children with ASD in the dental setting [25] (Table 3).

For the NRSI, it was rare for the overall judgement of bias to be low due to confounding. For this review, we accepted the outcomes at all levels from all the included papers, unless the paper did not show sufficient ability to produce a valid conclusion.

There are several limitations to this study. Most of the included studies had a small sample size hence may not be able to fully demonstrate the optimal benefit of specific behavioural strategies on the children from compared groups. Furthermore, some studies lacked control groups. Qualitative assessment could also benefit from the studies in addition to qualitative parameters measured to provide in-depth response on behavioural modification effects [37–39].

## Conclusion

This systematic review provided current available approaches yet inconclusive evidence on the effectiveness of the psychological approach for managing children with ASD at dental setting. Although the impact of the approach on the management of dental anxiety, the level of children’s cooperation, and the success of the implementation of dental procedures was reported, the study design of these behavioural modification techniques requires better randomisation and bias control to suggest effectiveness of intervention.

## Data Availability

All data generated or analysed during this study are included in this published article. Additional data is available from the corresponding author on reasonable request.
